# Differential regulation of morphine antinociceptive effects by endogenous enkephalinergic system in the forebrain of mice

**DOI:** 10.1186/1744-8069-4-41

**Published:** 2008-09-30

**Authors:** Tsung-Chieh Chen, Ying-Ying Cheng, Wei-Zen Sun, Bai-Chuang Shyu

**Affiliations:** 1 Institute of Biomedical Sciences, Academia Sinica, Taipei, 11529, Taiwan, ROC; 2 Department of Anaesthesiology, National Taiwan University Hospital, Taipei, 10002, Taiwan, ROC

## Abstract

**Background:**

Mice lacking the preproenkephalin (*ppENK*) gene are hyperalgesic and show more anxiety and aggression than wild-type (WT) mice. The marked behavioral changes in *ppENK *knock-out (KO) mice appeared to occur in supraspinal response to painful stimuli. However the functional role of enkephalins in the supraspinal nociceptive processing and their underlying mechanism is not clear. The aim of present study was to compare supraspinal nociceptive and morphine antinociceptive responses between WT and *ppENK *KO mice.

**Results:**

The genotypes of bred KO mice were confirmed by PCR. Met-enkephalin immunoreactive neurons were labeled in the caudate-putamen, intermediated part of lateral septum, lateral globus pallidus, intermediated part of lateral septum, hypothalamus, and amygdala of WT mice. Met-enkephalin immunoreactive neurons were not found in the same brain areas in KO mice. Tail withdrawal and von Frey test results did not differ between WT and KO mice. KO mice had shorter latency to start paw licking than WT mice in the hot plate test. The maximal percent effect of morphine treatments (5 mg/kg and 10 mg/kg, i.p.) differed between WT and KO mice in hot plate test. The current source density (CSD) profiles evoked by peripheral noxious stimuli in the primary somatosenstory cortex (S1) and anterior cingulate cortex (ACC) were similar in WT and KO mice. After morphine injection, the amplitude of the laser-evoked sink currents was decreased in S1 while the amplitude of electrical-evoked sink currents was increased in the ACC. These differential morphine effects in S1 and ACC were enhanced in KO mice. Facilitation of synaptic currents in the ACC is mediated by GABA inhibitory interneurons in the local circuitry. Percent increases in opioid receptor binding in S1 and ACC were 5.1% and 5.8%, respectively.

**Conclusion:**

The present results indicate that the endogenous enkephalin system is not involved in acute nociceptive transmission in the spinal cord, S1, and ACC. However, morphine preferentially suppressed supraspinal related nociceptive behavior in KO mice. This effect was reflected in the potentiated differential effects of morphine in the S1 and ACC in KO mice. This potentiation may be due to an up-regulation of opioid receptors. Thus these findings strongly suggest an antagonistic interaction between the endogenous enkephalinergic system and exogenous opioid analgesic actions in the supraspinal brain structures.

## Background

Opioid systems play an important role in numerous functions in the central nervous system (CNS) including pain modulation, stress-induced analgesia, reproductive activities, drinking, learning, motor behavior, mental illness and mood [1,2]. Endogenous opioid peptide precursors expressed in neurons are enzymatically cleaved to produce enkephalin, dynorphins and *β*-endorphin. Cleavage of the pre-proenkephalin (*ppENK*) precursor yields met-enkephalin and leu-enkephalin which are endogenous ligands of the *μ*- and *δ*-opioid receptors [3]. Evidence has indicated that the endogenous enkephalinergic system is involved in the antinociceptive response. For example, oral administration of BL-2401 (inhibitor of the enkephalin-catabolizing enzyme) and RB101 and SCH-32615 (enkephalinase inhibitors) to mice induces a strong, naloxone-reversible antinociceptive response [4-6]. Intrathecal administration of DAMPGO and DPDPE produce antinociception via an interaction with spinal opioid *μ*- and *δ*-receptors [7]. Also, an antinociceptive effect was prevented in mice pre-treated intrathecally with met-enkephalin antiserum [8].

The role of endogenous opioid peptides has recently been investigated using knockout (KO) mice [9]. Pre-proenkephalin deficient mice are healthy but display significant behavioral abnormalities. Increased anxiety and offensive aggressiveness is observed in male [9] and female [10]mice. In behavioral tests, the *ppENK *KO exhibit more exaggerated responses to painful stimuli than control wild-type mice (WT). Furthermore, nicotine-induced antinociception is decreased in mice lacking the *ppENK *gene [9,11,12]. The marked behavioral changes in KO mice appear to occur via the supraspinal response to painful stimuli [9]. However, the functional role and mechanism of action of enkephalin in supraspinal nociceptive processing is unclear.

The primary somatosensory cortex (S1) and anterior cingulate cortex (ACC) are two important supraspinal brain regions mediating discriminative and affective aspects of pain responses respectively [13-16]. Unit activities and extracellular field potentials evoked by noxious stimulation of cutaneous tissue have been used for investigation of nociceptive information processing within the S1 [17-19] and ACC [20-22]. Administration of morphine caused a concomitant reduction in the amplitude of noxious-evoked field potentials, ensemble neuronal unit activities and evoked synaptic currents recorded in the S1 [19,23,24]. These effects were reversed by naloxone treatment. The effect of morphine could be induced locally by topical application of morphine to the cortex, resulting in significant decreases in the pain intensity rating [25]. Furthermore, in an experiment examining the co-registration of noxious-evoked ensemble unit activities in the S1 and ACC of behaving rats, a single dose of morphine intraperitoneally suppressed the long latency response in the S1 and significantly attenuated early and late responses in the ACC [26]. However, it is still unclear whether deficiencies in the endogenous enkephalin system have any differential effects on supraspinal pain processing.

Evoked extracellular field potentials in the cortex are commonly taken as a measure of nociceptive input to the local region. However, the interpretation of extracellular field potential data has inherent ambiguity [27]. The current source density (CSD) method can be used to accurately localize the synaptic action by input signals from the S1 and ACC. Extracellular field potentials measured by multi-channel probes can provide information regarding the active processes of ionic flow into and out of cells that generate postsynaptic potentials. The CSD method can be used to provide sink and source information from such measurements. We have previously measured synaptic currents activated by CO_2 _laser pulses in the S1 or noxious electrical pulses in the ACC using multichannel probes and CSD analysis [19,21]. The evoked CSD profiles and varied stimulation methods allowed us to examine the temporal and spatial processing of nociceptive synaptic transmission in the intracortical regions.

To investigate the role of the endogenous enkephalin opioid system in the supraspinal nociceptive response, we used *ppENK *knockout (KO) mice. We examined the effect of endogenous enkephalin deficiency on nociception by tail-withdrawal, hot-plate and von Frey behavior tests and the effect of morphine. We characterized and compared evoked synaptic currents in the S1 and ACC of WT and KO mice in response to noxious stimuli. The differential effects and underlying mechanism of exogenous morphine treatment in enkephalin deficient mice was further examined.

## Results

### Verification of enkephalin deficiency in KO mice

**Figure 1:**
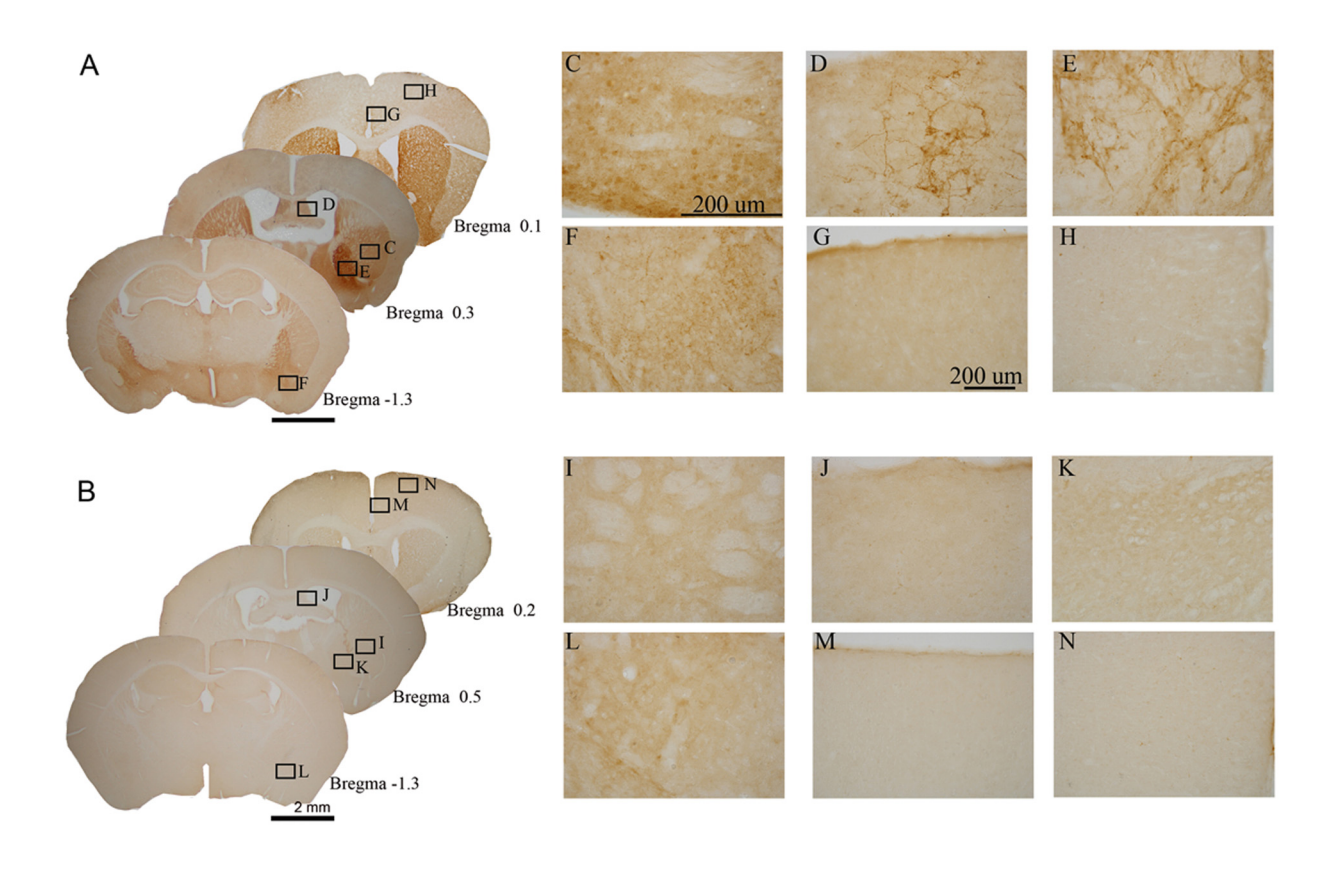
**Comparison of the distributions and densities of immunoreactive enkephalin neurons and fibers in WT and KO mice.** Low magnification photomicrography of coronal brain sections with immunostaining of enkephalinergic neurons and fibers in WT (A) and KO (B) mice. (C) – (N) show higher magnification photomicrography of immunostained brain regions of WT mice (C – H) and KO mice (I – N) enlarged from square areas indicated in A and B respectively. The brain regions magnified are: (C), (I) lateral globus pallidus, (D), (J) lateral septum, (E), (K) intermediated part of caudate putamen, (F), (L) amygdaloid nuclear region, (G), (M) cingulate cortex and (H), (N) sensory motor cortex.

Immunohistochemical labeling of enkephalin fiber terminals was performed to confirm enkephalin deficiency in KO mice. Qualitative changes in the labelling of terminals in different brain regions were observed in low magnification microscope images (Figure 1A, WT and B, KO; serial coronal sections). In WT mice, met-enkephalin immunoreactive cell bodies were observed in the lateral globus pallidus (Figure 1C) and met-enkephalin immunoreactive cell bodies and fibers were labelled in the intermediated part of the lateral septum (Figure 1D), caudate putamen (Figure 1E), amygdaloid nuclei (Figure 1F). Less labelling with the met-enkephalin antibody was detected in the cingulate cortex (Figure 1G) and sensory motor cortex (Figure 1H) of WT mice. Met-enkephalin immunoreactivity in these regions was barely observed in corresponding brain sections of KO mice (Figure 1I–N). The immunohistochemical labelling was consistent in all WT (n = 9) and KO (n = 6) mice examined.

### Behavioral changes and effect of morphine on supraspinal nociceptive responses

**Figure 2:**
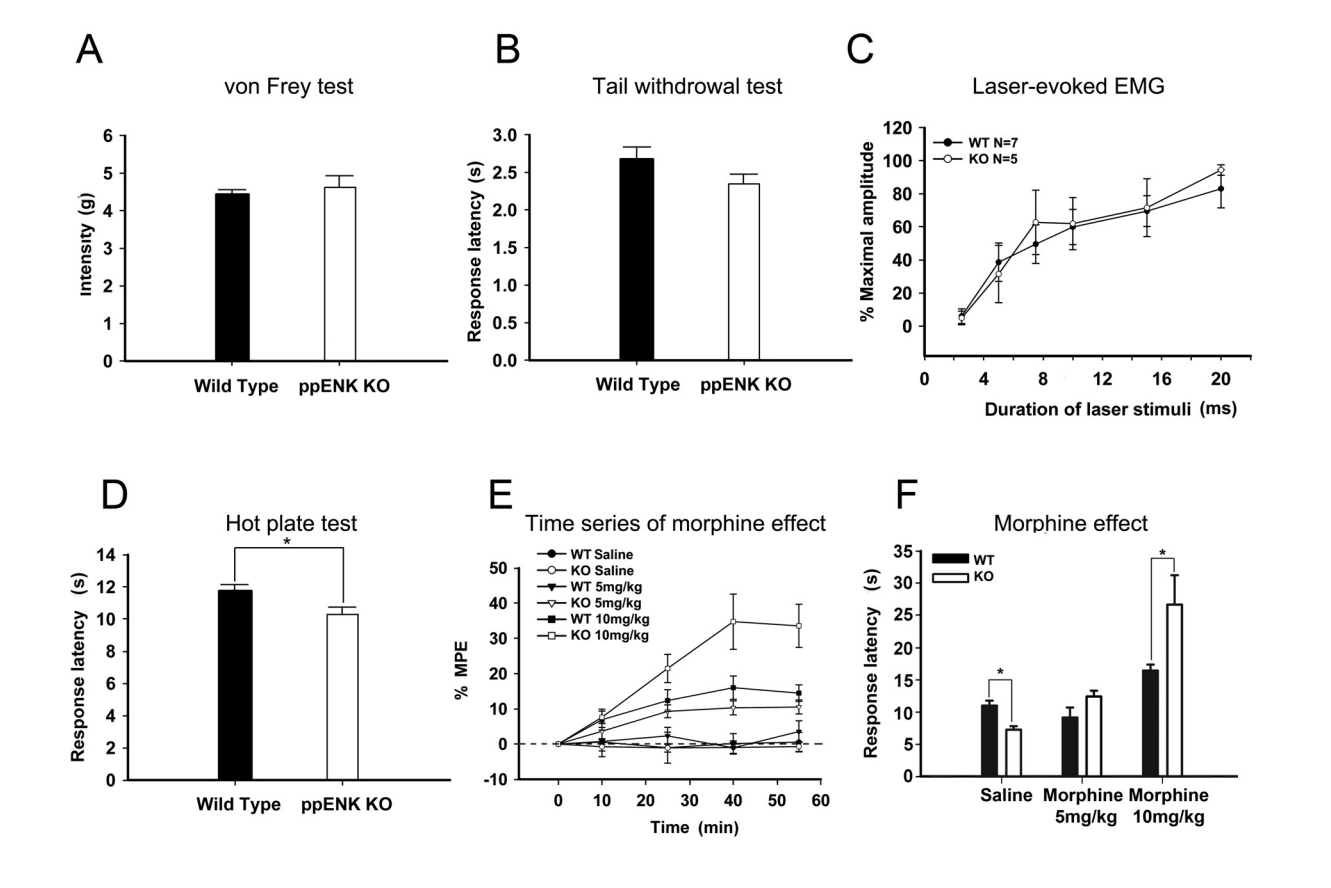
**Comparison of nociceptive behavioural responses and the antinociceptive effect of morphine in WT and KO mice.** Results from the von Frey test (A) tail withdrawal test (B), EMG evoked by incremental laser pulse duration (C), and hot plate test (D) are shown. The analgesic effects of morphine (5 mg/kg and 10 mg/kg) on the hot plate test as a function of time (E) and 40 min after morphine administration are also shown (F).

Von Frey and tail withdrawal tests were carried out to test the spinal nociceptive behaviors in WT and KO mice. No significant differences were found in the paw withdrawal intensity of the von Frey test or the latencies of the tail withdrawal test in WT (von Frey test, n = 9; withdrawal test, n = 8) and KO (von Frey test, n = 9; withdrawal test, n = 17) mice (Figure 2A and 2B). To further test the spinal withdrawal reflexes, hind limb electromyograms (EMG) evoked by laser stimuli at the hind paw were measured and compared in WT and KO mice. The amplitudes of EMG evoked by incremented laser stimulus duration were normalized to the maximal response. The trend of amplitude increases did not differ between WT (n = 7) and KO (n = 5) mice (Figure 2C). The peak amplitudes of EMG evoked at the 20 ms laser pulse duration were not significantly different for WT or KO mice (WT: 0.0334 ± 0.018 mV, n = 7; KO: 0.0287 ± 0.001 mV, n = 5).

In the hot plate test, the latency to the first sign of paw licking in KO mice was significantly shorter than in WT mice (p = 0.012, WT: n = 27; KO: n = 43, Figure 2D). The effect of morphine on supraspinal nociceptive behavior was further examined using the hot plate test. The analgesic effect of morphine was maximal at 40 min after the morphine injection and was dose-dependent (5 mg/kg, WT: n = 10; KO: n = 7, 10 mg/kg, WT: n = 9; KO: n = 9, saline, WT: n = 9; KO: n = 6) (Figure 2E). The percentage of the maximum possible effect (% MPE) at 40 min was significantly higher in KO mice than WT mice after both dosages of morphine (5 mg/kg, p = 0.049, and 10 mg/kg, p = 0.007, Figure 2F).

### Evoked responses in S1 following laser stimulation

**Figure 3:**
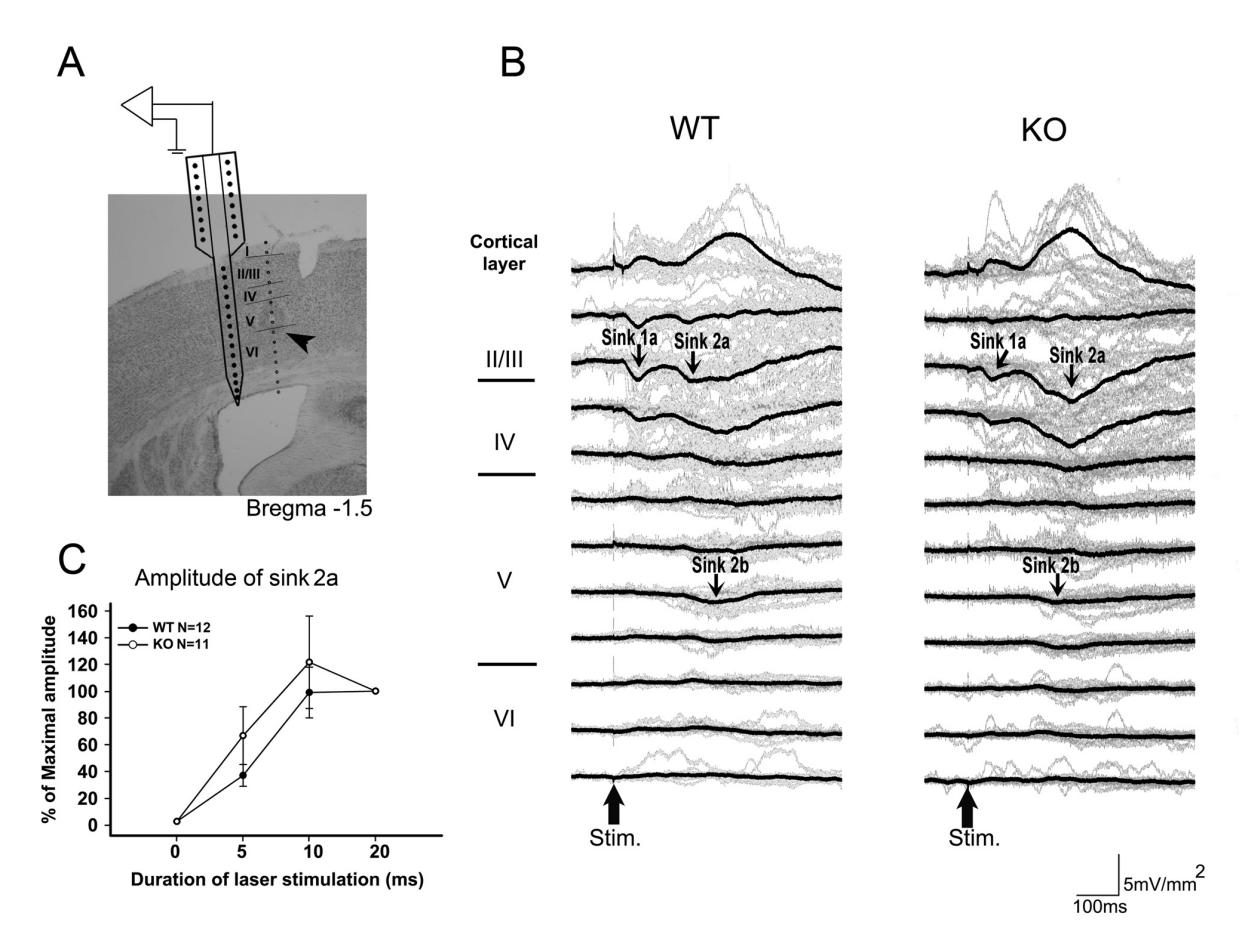
**Laser evoked CSD profiles across cortical layers of the S1 in WT and KO mice.** (A) Schematic diagram of the recording scheme. The position of the multichannel probe is overlaid with histological sections from the S1. Cortical layers are indicated by roman numerals. Arrow indicates the electrolytic lesion mark in layer V. (B) CSD sweeps across the cortical layers in WT (left panel) and KO (right panel) mice. Gray lines indicate the averaged CSD sweeps from individual mice. Black lines indicated the grand averaged CSD sweeps from WT (n = 18) and KO (n = 16) mice. Sink currents are in the downward direction and source currents are in the upward direction. Sink 1a (early component) and sink 2a and sink 2b (late components) were identified. All of the evoked CSD profile was evoked by laser stimuli with intensity of 10 W and duration of 20 ms. (C) Percent of maximal amplitude change of sink 2a evoked by increment of laser duration.

A schematic diagram depicting recording in the S1 by a 16 channel Michigan probe is shown in Figure 3A. The CSD was calculated from multichannel cortical field potentials recorded in the contralateral S1 evoked by laser stimulation (10 W, 5~20 ms duration) of the left hind paw. Evoked responses were reproducible and consistent in individual animals. Grand averaging was used to average the evoked responses obtained from different mice, as described in previous work [19]. The CSD profiles across the depth of cortical layers in WT (n = 18) and KO (n = 16) mice following laser stimulation are shown in Figure 3B. Two major groups of sink currents (sink 1a and sink 2a &b) were detected in layers II/III, IV and V of the S1 by laser stimuli with 10 W and 20 ms duration. This finding was consistent with our previous results in rats. Therefore, these two distinct sink current components likely represent the cortical responses of A-delta and C-fiber activation [19]. The latency and amplitudes of the laser evoked prominent sink currents in WT and KO mice are listed in Table 1. The amplitude of the laser evoked sink 1a current at 20 ms laser pulse duration was -3.37 ± 0.48 mV/mm^2 ^(n = 18) in WT mice and was significantly different from that evoked in KO mice (-1.60 ± 0.52 mV/mm^2^, n = 16). The amplitudes of the major group of sink currents (sink 2a) increased with increasing laser pulse duration (Figure 3C). The amplitudes of sink 2b sink currents were not significantly different between WT and KO mice.

**Table 1:** Amplitudes and latencies of laser-evoked sink currents in the S1 of WT and KO mice.

	**Amplitude (mV/mm^2^)**			**Latency (ms)**		
	**Sink 1a**	**Sink 2a**	**Sink 2b**	**Sink 1a**	**Sink 2a**	**Sink 2b**

**WT (n = 18)**	-3.37 ± 0.40*	-5.99 ± 0.70	-1.60 ± 0.10	56.82 ± 3.00	212.46 ± 14.10	270.40 ± 10.80
**KO (n = 16)**	-1.60 ± 0.50	-5.53 ± 0.70	-1.34 ± 0.10	57.19 ± 2.80	211.14 ± 11.20	225.24 ± 12.00

* p = 0.009, Sink 1a of WT v.s. Sink 1a of KO

### Effects of morphine on laser evoked CSD profile and sink current components in the S1

**Figure 4:**
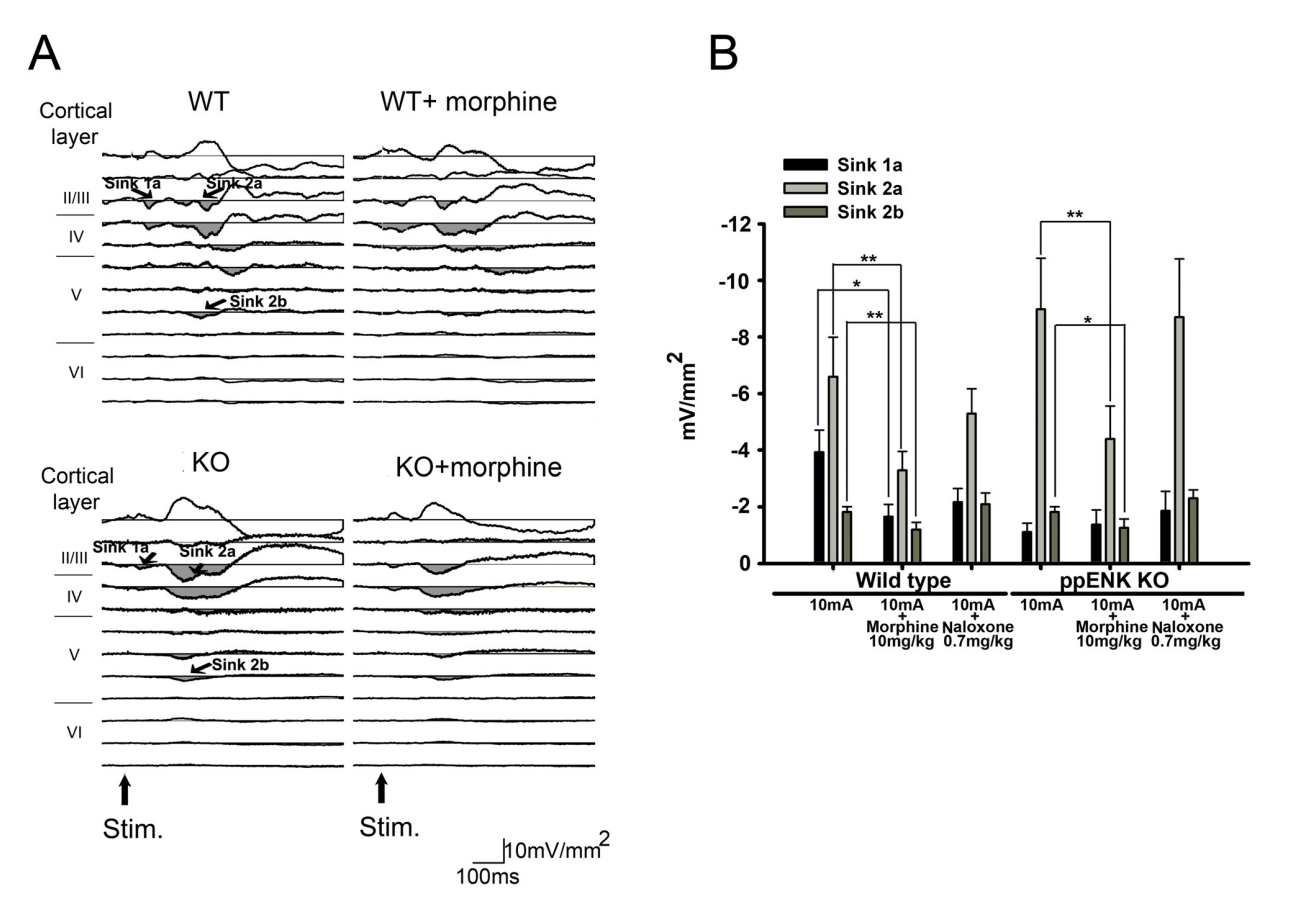
**Effect of morphine on the sink current evoked in the S1 by laser pulses (10 W and 20 ms duration).** (A) Example of laser evoked CSD profiles in the S1 before and after morphine treatment (10 mg/kg) in WT (upper panel) and KO (lower panel) mice. (B) Statistical analysis of the effect of morphine on sink 1a, sink 2a and sink 2b in WT and KO mice. Reversibility of the effect was evaluated by treatment with naloxone (0.7 mg/kg). * p < 0.05. ** p < 0.01.

The effect of morphine on laser evoked CSD profiles was tested after stable evoked cortical responses were obtained. Amplitudes of evoked sink currents were reduced after 10 mg/kg morphine treatment in both WT and KO mice. Typical examples of the effect of morphine on CSD profiles in S1 in both WT and KO mice are shown in Figure 4A. This result is consistent with our previous findings [19]. The amplitude of the sink 2a current was reduced by 66.11 ± 4.52% (10 mg/kg morphine) in WT mice (n = 8). The suppressive effect was also evident in KO mice (n = 9) where a 52.31 ± 4.13% (10 mg/kg morphine) reduction was observed. Statistical analysis of the morphine effect on the amplitudes of sink currents is shown in Figure 4B. The suppressive effect of morphine was significant in sink 2a and sink 2b currents. The effect of morphine in both WT and KO mice was reversed by treatment with naloxone (0.7 mg/kg).

### Evoked responses in ACC following electrical stimulation at right hind paw

**Figure 5:**
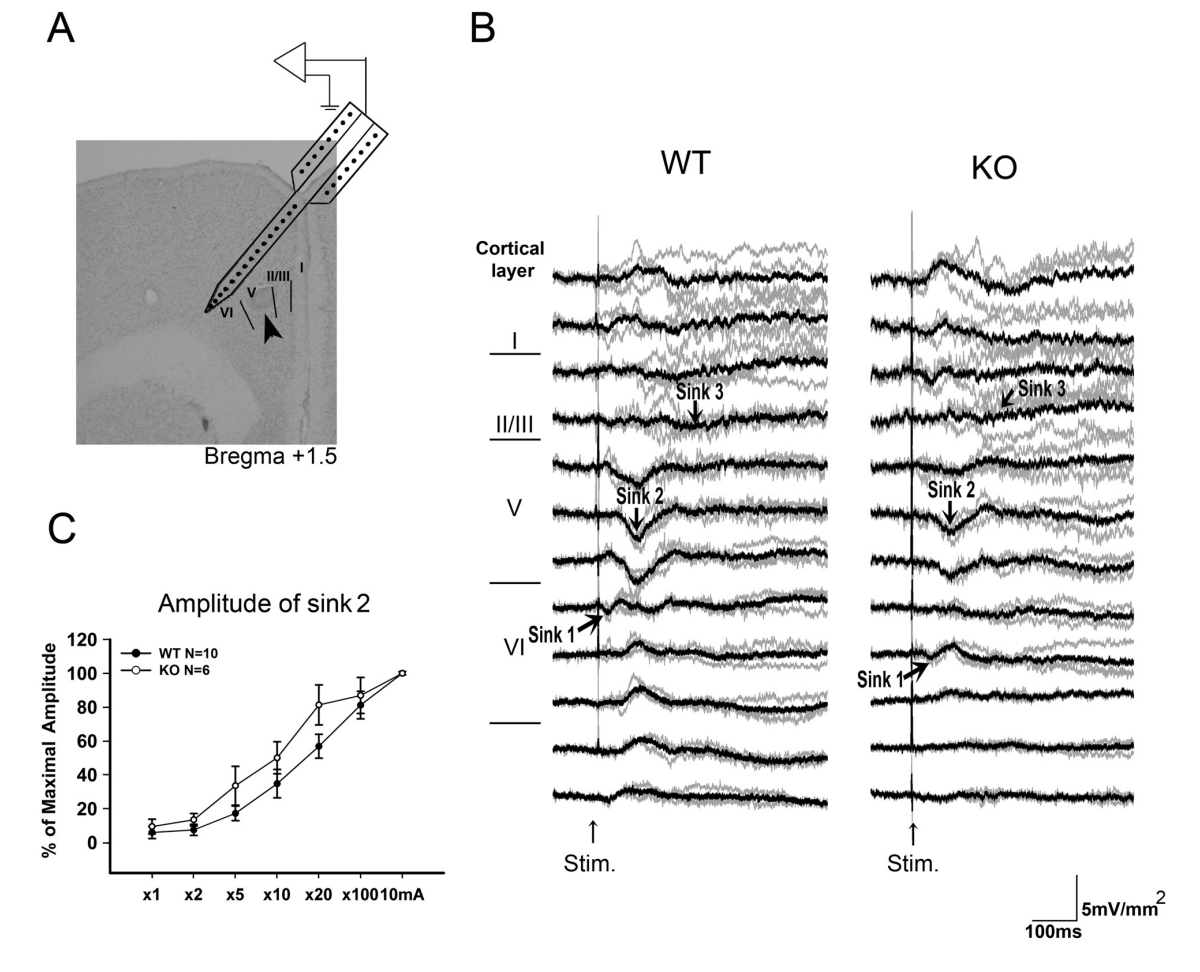
**Electrical evoked CSD profiles across cortical layers of the ACC in WT and KO mice. **(A) Schematic diagram of the recording scheme and the electrical stimulation parameters: 10 mA, 0.5 ms duration and 0.1 Hz. The position of the multichannel probe is overlaid with histological sections of the ACC. Cortical layers are indicated by roman numerals. Arrow indicates the electrolytic lesion mark in layer V. (B) CSD sweeps across the cortical layers in WT (left panel) and KO (right panel) mice. Gray lines indicate averaged CSD sweeps from individual mice. Black lines indicate the grand averaged CSD sweeps from WT (n = 4) and KO (n = 4) mice. Sink 1, sink 2 and sink 3 were identified. (C) Percent of maximal amplitude change of sink 2 evoked by increment of electrical intensity of stimulus.

The multichannel electrode and recording scheme in the ACC area are illustrated in Figure 5A. The grand average of CSD sweeps evoked by electrical stimulation of 10 mA, 0.5 ms duration and, 0.1 Hz in the hind paw of WT (n = 4) and KO (n = 4) is shown in Figure 5B. An early small sink current appeared in upper layer VI. A second prominent sink current, sink 2, was evoked at layer V. A third sink current was evoked at a longer latency in layer II/III. Amplitudes and latencies of these sink components are listed in Table 2. Amplitude of the sink currents increased with increasing stimulation intensity in WT and KO mice (sink 2, Figure 5C). The amplitude of the sink 2 current evoked at 10 mA in KO mice was not significantly different from that evoked in WT mice (WT: -0.84 ± 0.10 mV/mm^2^, n = 10; KO: -0.91 ± 0.05 mV/mm^2^, n = 6).

**Table 2:** Amplitudes and latencies of noxious electrically-evoked sink currents in the ACC of WT and KO mice.

	**Amplitude (mV/mm^2^)**			**Latency (ms)**		
	**Sink 1**	**Sink 2**	**Sink 3**	**Sink 1**	**Sink 2**	**Sink 3**

**WT (n = 18)**	-0.32 ± 0.08	-0.83 ± 0.11	-0.56 ± 0.12	27.72 ± 0.12	95.17 ± 13.50	192.10 ± 14.10
**KO (n = 16)**	-0.31 ± 0.20	-0.91 ± 0.10	-0.66 ± 0.20	38.70 ± 2.30	91.39 ± 6.40	190.60 ± 17.90

### The effect of morphine on evoked CSD profiles in the ACC

**Figure 6:**
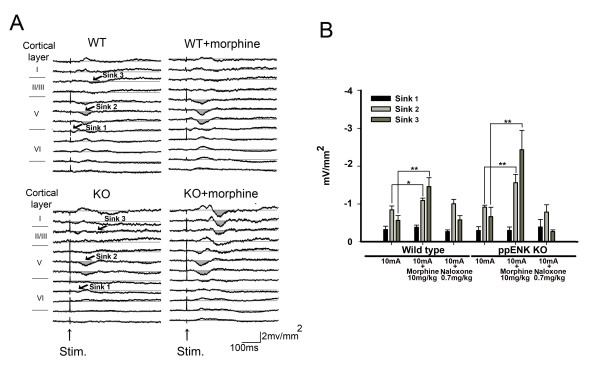
**Effect of morphine on the sink current in the ACC evoked by high electrical intensity stimulation.** (A) Example of CSD profiles evoked by electrical stimulation (10 mA, 0.5 ms duration and 0.1 Hz) in the hind paw before and after morphine treatment (10 mg/kg) in WT (upper panel) and KO (lower panel) mice. (B) Statistical analysis of the effect of morphine on sink 1, sink 2 and sink 3 in WT and KO mice. Reversibility of the effect was evaluated by treatment with naloxone (0.7 mg/kg). * p < 0.05. ** p < 0.01.

We have found previously that current sinks evoked by peripheral noxious stimuli in rats are potentiated by morphine treatment [21]. Enhancement of the evoked sink current, induced by 10 mA, 0.5 ms duration, 0.1 Hz electrical stimulation in the hind paw, was confirmed in the present study in both WT and KO mice. A typical example is shown in Figure 6A. Statistical analysis of the effect of morphine on the sink current components is shown in Figure 6B. Sink 2 and sink 3 currents were significantly enhanced by morphine (10 mg/kg). After morphine injection, there was a 137.70 ± 11.35% and 278.41 ± 55.90% increase of sink 2 and sink 3 currents respectively in WT mice, whereas the increase of sink 2 and sink 3 currents in KO mice was 183.45 ± 25.87% and 365.34 ± 103.78% respectively. The effect of morphine in both WT and KO mice was reversed by naloxone (0.7 mg/kg).

### Effect of morphine on simultaneously recorded responses in the S1 and ACC

**Figure 7:**
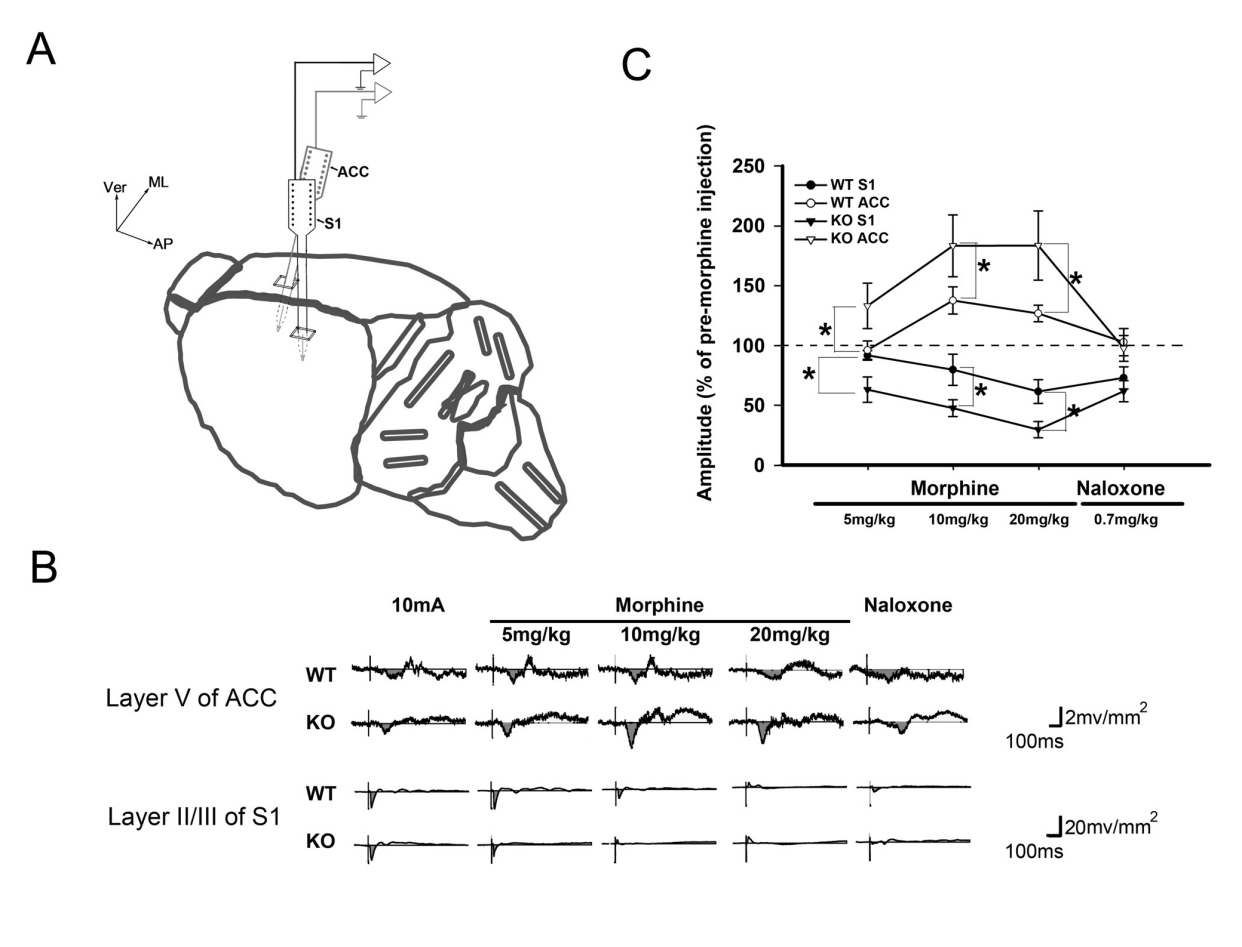
**Effect of morphine on simultaneously recorded evoked cortical responses in the S1 and ACC.** (A) Schematic diagram of the recording scheme of the multichannel probes placed in the S1 and ACC regions. (B) Example sweeps of sink currents in the ACC layer V (upper panel) and S1 layer II/III (lower panel) evoked by the high intensity electrical stimulation (10 mA, 0.5 ms duration and 0.1 Hz) in the hind paw. Example sweeps of evoked cortical sink currents after morphine treatment (5 mg/kg, 10 mg/kg and 20 mg/kg) and reversal by naloxone treatment were demonstrated. (C) Statistical analysis of the effect of morphine on simultaneously recorded S1 and ACC evoked sink currents. Data are presented as mean ± SEM. Amplitude of the sink current after morphine injection is expressed as a percentage of the amplitude of the sink current measured before morphine injection. * p < 0.05.

Morphine evoked opposite responses in the ACC and S1 when recording measurements were made separately. This raised the question whether the responses were due to morphine or variations in the sampling condition. Therefore, the effect of morphine on the evoked responses in the ACC and S1 was examined simultaneously as shown with the placement of recording probes in Figure 7A. In our pilot study, we found that the responses in the ACC evoked by laser pulses were too small and variable to evaluate reliably. In order to obtain strong, reliable responses for comparison between the ACC and S1, we used strong electrical stimuli, 10 mA, 0.5 ms duration and 0.1 Hz, as the noxious stimulation. An early and marked sink current was evoked in layer IV of S1 at 20.72 ± 1.57 ms with an amplitude of -18.99 ± 4.163 mV/mm^2 ^after electrical stimuli. The stimulating thresholds for evoking sink currents ranged from 0.03 to 0.12 mA in WT mice and from 0.06 to 0.18 mA in KO mice. Prominent sink current components in layers II/III and V of S1 (Table 3) and layer V of ACC could be evoked by intense electrical currents (10 mA) applied at the hind paw and recorded simultaneously (Figure 7B). Typical effects of morphine (5 mg/kg, 10 mg/kg and 20 mg/kg) are shown in Figure 7B.

**Table 3:** Amplitudes and latencies of noxious electrically-evoked sink currents in the S1 of WT and KO mice.

	**Amplitude (mV/mm^2^)**			**Latency (ms)**		
	**Sink 1**	**Sink 2**	**Sink 3**	**Sink 1**	**Sink 2**	**Sink 3**

**WT (n = 18)**	-18.99 ± 4.10	-2.80 ± 0.40	-4.14 ± 0.80	20.72 ± 1.50	39.26 ± 2.50	96.30 ± 6.10
**KO (n = 16)**	-23.10 ± 5.20	-2.78 ± 0.30	-4.06 ± 0.90	20.05 ± 1.30	39.53 ± 2.90	90.30 ± 3.60

With 5 mg/kg morphine, the amplitude of sink 2 in the ACC evoked by electrical stimulation was 96.2 ± 7.6% of the control amplitude in WT mice (n = 8) and 133.2 ± 18.9% of the control amplitude in KO mice (n = 9). Morphine decreased the sink current in layer II/III in the S1 to 91.8 ± 4.0% of the control amplitude in WT mice (n = 8) and 63.1 ± 10% of the control amplitude in KO mice (n = 9).

With 10 mg/kg morphine, the amplitude of the sink current in the ACC increased to 137.3 ± 11.3% of the control amplitude in WT mice (n = 9) and 183.4 ± 25.3% of the control amplitude in KO mice (n = 7). In the S1, morphine decreased the sink current evoked by electrical stimulation to 79.7 ± 13.0% of the control amplitude in WT mice (n = 9) and 47.7 ± 7.0% of the control amplitude in KO mice (n = 7).

With 20 mg/kg morphine, the amplitude of the sink current in the ACC increased to 126.9 ± 6.8% of the control amplitude in WT mice (n = 8) and 183.7 ± 28.9% of the control amplitude in KO mice (n = 6). In the S1, morphine decreased the CSD profile evoked by electrical stimulation to 61.5 ± 9.9% of the control amplitude in WT mice (n = 8) and 29.7 ± 6.7% of the control amplitude in KO mice (n = 6). The effect of morphine was reversed in both the S1 and ACC by intraperitoneal injection of naloxone (0.7 mg/kg).

### Effect of GABA_B _agonist and antagonist on evoked CSD responses in the ACC

**Figure 8:**
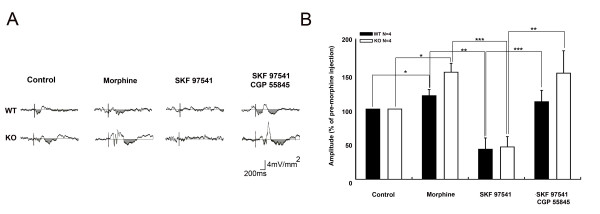
**Effect of GABA_B _agonist and antagonist on the evoked sink currents in the cortical layer V of the ACC.** (A) The electrical stimulation (10 mA, 0.5 ms duration and 0.1 Hz) was applied in the right paw of the mice. Example sweeps of evoked sink currents in layer V of the ACC under before and after treatment with morphine, SKF 97541 and CGP55845. (B) Statistical analysis of the effects of morphine, SKF 97541 and CGP55845 on the layer V sink current in the ACC. * p < 0.05. ** p < 0.01.

The enhancing effect of morphine on CSD profiles in the ACC has been investigated previously in rats [21]. We have previously shown that the effect of morphine is mediated by local opioid interneurons. Several reports have implicated GABAergic interneurons in opioid local action in ACC circuitry [28,29]. To further examine the role of GABAergic interneurons in the effect of morphine, the GABA_B _receptor agonist; SKF 97541, and GABA_B _receptor antagonist; CGP 55845, were administered after morphine injections in WT and KO mice. Example sweeps of sink 2 current in layer V of the ACC were averaged from 20 sweeps of cortical response following electrical stimulation of the hind paw with 10 mA, 0.5 ms duration and 0.1 Hz (Figure 8A). Morphine (10 mg/kg) significantly increased the evoked CSD response (sink 2) in layer V of the ACC (Figure 8A &8B, KO mice, n = 4, p = 0.01; WT mice, n = 4, p = 0.05). Morphine induced potentiation of the evoked CSD response (sink 2) in the ACC was decreased after 30 min of 0.3 mg/kg SKF 97541 treatment (KO: n = 4, p = 0.005; WT: n = 4, p = 0.003). The inhibitory effect of SKF 97541 on the evoked response in the ACC was reversed by 10 mg/kg CGP 55845 (KO: n = 4, p = 0.02; WT: n = 4, p = 0.0001).

### Alteration of *μ*-opioid receptor responses in the ACC and S1

**Figure 9:**
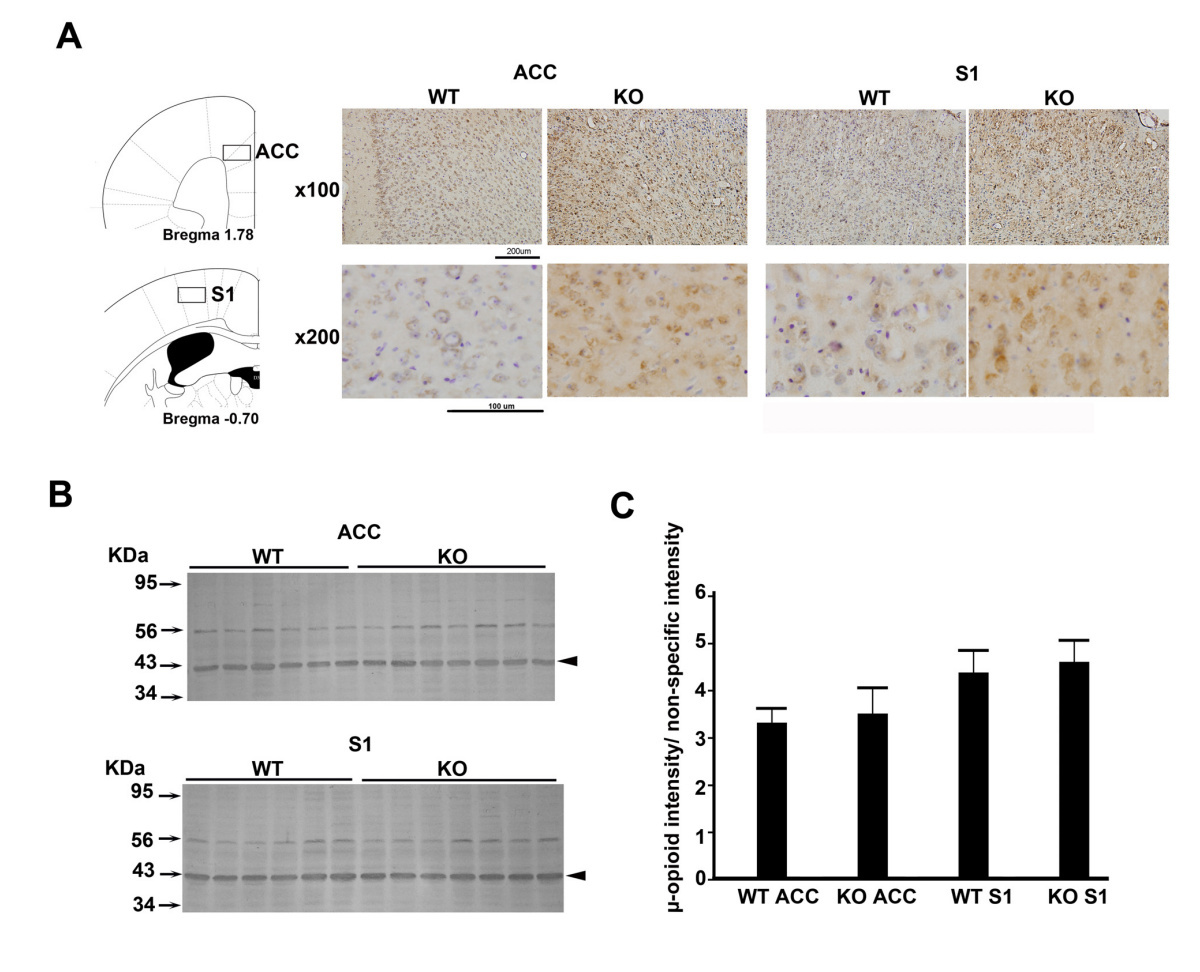
**Detection and measurement of *μ*-opioid receptors by immunostaining and Western blotting.** (A) Immuoreactive *μ*-opioid-receptors were stained in the S1 and ACC of WT and KO mice. Micrographs of *μ*-opioid-receptors (100× magnification) are shown in the upper panel. Micrographs at higher magnification are shown in the lower panel. Locations that were immunopositive for *μ*-opioid-receptors are indicated by square boxes in the left panel. (B) Western blot data provided a quantitative measurement of *μ*-opioid receptors in the cortical region of WT and KO mice. The *μ*-opioid receptor band is located at 43 kDa (arrow heads). Non-specific binding was used as an internal control (56 kDa). (C) The relative quantities of *μ*-opioid receptor were measured by taking the ratio of specific and non-specific binding in the S1 and ACC of WT (n = 6) and KO (n = 7) mice. Data are presented as the mean ± SEM.

Morphine enhanced both the excitatory effect in the ACC and suppressive effect in the S1 in KO mice. It is unlikely that these enhancements are due to the endogenous release of enkephalin because the deficiency of enkephalin in fiber terminals was demonstrated for KO mice. A likely explanation is up-regulation of opiod receptors as reported previously [30]. *μ*-opioid receptor immunoreactivity in the ACC and S1 is shown in Figure 9A. The distribution and density of *μ*-opioid receptors in the ACC and S1 are qualitatively different in WT (n = 8) and KO (n = 8) mice. The ratio of specific and non-specific binding at opioid receptors was compared using Western blot data (Figure 9B). The difference in ratio in WT (n = 6) and KO (n = 7) mice was not statistically different but there is a trend that receptor binding in the ACC and S1 is higher in KO mice than WT mice. The receptor binding from KO mice was 105.8% that of receptors from WT mice in the ACC and 105.1% of WT mice in the S1.

## Discussion

The present study revealed that *ppENK *deficiency mainly influenced the nociceptive behavioral responses manifested at the supraspinal level. The antinociceptive effect of morphine was dose-dependent in WT mice. The effect of morphine was enhanced in KO mice as shown by the hot plate test. Acute nociceptive responses in the cortical regions indicated by alteration of CSD profiles did not differ in KO mice compared to WT mice, indicating that the enkephalin system is not directly involved in acute and phasic nociceptive transmission. Systemic morphine treatment resulted in opposing effects on noxiously evoked synaptic responses in the S1 and ACC. The effect of morphine in both regions was significantly enhanced in KO mice. This result strongly indicates the potential antagonistic interaction between endogenous enkephalinergic system and exogenous opioid analgesic mediation in the supraspinal region. Enhancement of the effect of morphine may be due to up-regulation of *μ*-opioid receptor expression when enkephalin release is deficient. Morphine-enhanced nociceptive synaptic responses in the ACC were blocked by a GABA_B _receptor agonist indicating that the endogenous enkaphalinergic system is involved in the anti-nociceptive response through the inhibitory GABA_B _receptor.

### Effect of *ppENK *deficiency on supraspinal pain behavior

*ppENK *deficient (KO) mice exhibit normal pain responses in von-Frey and tail-withdrawal tests and similar leg withdrawal EMG activities as WT mice. Increased pain sensitivity in KO mice was only observed in the hot-plate test. In the hot plate test, the nocifensive behavior involved licking, flinching and head, trunk and limb coordination. Compared to the spinal reflexive behaviors measured by the von Frey and tail withdrawal tests, these behaviors are more complex, organized and unlearned behaviors and involve purposeful actions requiring supraspinal sensory processing [31]. This finding was consistent with a previous study in which *ppENK *KO mice expressed sensitive nociceptive responses to supraspinal behavior tests [9,10,12,32]. KO mice also displayed reduced exploratory activity in an unfamiliar environment [9]. Evidence indicated that brief exposure to short, emotionally-arousing, non-noxious stress, such as holding and novel environments, leads to an immediate and transient hyperalgesia [33-35]. It is unlikely that this effect resulted in the hyperalgesia observed in KO mice in the hot plate test. Firstly, WT and KO mice were exposed to the same testing environment and protocol. Secondly, restraint would have had a greater effect in the tail withdrawal test where the rats were held inside a cloth. However, the tail withdrawal test did not show any significant difference between WT and KO mice. Therefore, the hyperalgesia observed in KO mice can be attributed to the genetic factors leading to alteration of physiological responses, indicating that endogenous enkaphalins are involved in the modulation of nocifensive behavioral responses.

### Laser evoked CSD in the S1

Two distinct sink current components were observed in the S1 following brief CO_2 _laser pulses applied at the hind paw of WT and KO mice. The intracortical layer distribution and activation pattern of these two components were similar with those previously found in rats [19]. The early component of the laser-evoked responses observed in the present study was consistent with previously reported "laser-evoked potential (LEP)" findings [24,36,37] and the negative peak in intracortical recording following radiant heat stimulation of rat hind paw [38]. The cortical layer location and initiation timing of sink Ia suggest that this sink current is derived from a depolarization of the dendrites of spiny stellate neurons that receive inputs from specific thalamic afferents [39]. One important difference between the previous study in rats and the results in mice presented here is that the sink current activated in the deep layer was absent in mice. Also, the amplitude of the sink Ib current identified in rats was small. Deep cortical layers receive terminations from projection neurons of the posterior thalamic nucleus [40-42]. The thalamic afferent terminals in the deep cortical layers are sparse and not as dense as those in layer IV [42]. The synchronous, excitatory postsynaptic current in the deep layer would be more dispersed compared with that activated in the granular layer. Therefore, one possible reason that the sink current is absent in the deep layer is that the small amplitude, deep layer, early sink current in the early component was cancelled out in the grand averaged sweeps.

Nociceptive cortical responses following laser stimulation exhibited similar late onset latency of sink current in layer III–IV and layer V–VI as found previously in rats [19]. The sink source activation patterns and cortical layer distribution of the late component were similar to that of the early component. Thus, the excitatory synaptic events activated by the late onset thalamocortical afferents followed a similar intracortical layer-specific pathway to that in the early component. These early and late components are most likely multiple spinal pathways which transfer information from cutaneous nociceptive A-delta and C fibers to the SI in mice. However, similar amplitude of sink currents activated by graded stimuli in both WT and KO mice indicates that endogenous enkephalin peptide was not directly involved in normal nociceptive transmission.

### Electrical stimulation evoked CSD in the ACC

One early sink in layer V and two major sink currents in layer V and II/III were detected in the ACC following noxious electrical stimulation in both WT and KO mice. Although there was a difference in the layer distribution of the sink and source currents, similar evoked CSD profiles in the ACC were observed in rats in our previous study [21]. The earliest sink in layer VI, located near the layer V/VI border, has a shorter latency than that in layer II/III and likely reflects activation by thalamocortical afferent projections to cingulate neurons in deeper layers [43-46]. The early sink current in layer II/III was accompanied by a more superficial source current. This arrangement suggests that afferent fibers terminate on the spines of vertically oriented ascending dendrites of layer II/III pyramidal neurons [47], creating local sink currents and a distal source current in their apical dendrites [45,48,49]. Nociceptive-specific neurons in ACC layers V and VI have been described in several studies [24,50,51]. Strong activation of the layer II/III sink current by medial thalamus (MT) stimulation in previous studies suggests that sink currents in layer II/III may receive direct excitatory synaptic inputs from the MT [24,50,51]. Similar studies have demonstrated major thalamic projections to layers II/III and V in this brain region [43-45,52]. There was no significant difference in the evoked sink current in the ACC in WT and KO mice. Thereby indicating that nociceptive synaptic transmission in the thalamocingulate pathway is not mediated by the endogenous opioid system.

### Differential effect of morphine in the S1 and ACC

Morphine administration induced dose-dependent behavioral analgesia in both WT and KO mice. This antinociceptive effect was enhanced in KO mice. The analgesic effect was reflected in the suppressive effect of morphine on the activated, C-fiber related synaptic current in the S1 of WT mice. The enhanced antinociceptive effect during behavioral tests was also observed as a result of the suppression of evoked synaptic currents in the S1 of the KO mice. Systemic administration of morphine has been shown to produce analgesia at spinal and supraspinal levels [53,54] and C-fiber evoked ensemble neuronal activities are susceptible to morphine treatment [24]. However, we cannot exclude the possibility that this C-fiber response suppressive effect is exerted at the spinal level [23]. Direct evidence has shown that morphine may act directly and locally in the S1, as evidenced by suppression of the sensory rating in the formalin test or inhibition of noxiously-evoked neurotransmitter release [25,55].

Morphine resulted in enhancement effects in the ACC, in contrast to the suppressive effect observed in the S1. This finding is consistent with previous results in rats where peripheral noxious afferent-activated synaptic currents were enhanced following morphine treatment [21]. This effect was mediated by local intracortical circuitries. We have provided evidence of the involvement of GABA interneurons in this enhancement effect. The enhancement was attenuated by a GABA_B _receptor agonist. The effect of the GABA receptor agonist was reversed by a GABA_B _receptor antagonist, indicating that the response is specific to GABA_B _receptors. Evidence for morphine enhancement of excitatory synaptic transmission through inhibition of GABAergic interneurons has been found in the hippocampus and the periaqueductal grey matter [56-58]. In addition, the interactions between GABA and opioid receptor agonists in the antinociceptive effect and opioid-conditional response have been studied previously [28,59]. In the medial prefrontal cortex, thalamocingulate terminals make synapses on GABAergic interneurons as well as principal neurons [45]. This arrangement may enable GABAergic interneurons to inhibit cingulate principal neurons via feedforward inhibition. Moreover, the presence of GABAergic terminals, both pre- and post-synaptic from thalamocingulate synapses, may enable disinhibition of the interneurons following activation of thalmocingulate afferents [29,60].

### *μ*-Opioid receptor up-regulation in the cortical region of KO mice

Differential effects on the evoked synaptic currents in the S1 and ACC were produced by exogenous morphine treatment. The effect of morphine, whether suppressive or enhancing, was greater in KO mice than WT mice. The enhancement cannot be attributed to endogenous enkephalin release as the KO mice were shown to be deficient in enkephalin. Opioid receptor up-regulation has been observed when endogenous enkephalin release is deficient [30] or when mice were chronically treated with a morphine antagonist [61]. It has been suggested that proenkephalin peptides are tonically active at mu opioid receptors in brain regions where the receptors were up-regulated in the deficient state [30]. We found 5.8% and 5.1% increases in opioid receptor binding in the ACC and S1 respectively in the present study in KO mice compared to WT mice. Previous quantitative autoradiography studies to detect the level of opioid receptor subtypes in the brain of enkephalin knockout mice showed that the largest changes were observed in limbic regions. The percent increase of opioid receptor binding in KO mice is between 7.8 and 18% in the ACC [30]. Although our finding of receptor up-regulation is consistent with previous studies, there is a discrepancy between the percentage increase of the receptor up-regulation and the percentage changes in the behavioral scores and evoked cortical responses following morphine treatment. Thus, up-regulation of opioid receptors may only be partially responsible for the increase in sensitivity to exogenous morphine treatment and the potentiated morphine effect in both the S1 and ACC. Other mechanisms, such as the regulation of post-receptor signal transduction and the anti-opioid system, may also be involved [62,63]. Opioid receptor up-regulation can also be induced by chronic opioid antagonist treatment and it has been suggested that this regulation is associated with alteration of proteins involved in receptor trafficking, reducing constitutive internalization of opioid receptors [61]. Chronic opioid treatment not only impairs opioid receptor function but also alters G protein coupling events resulting from receptor activation. Qualitative changes of components of opioid receptor-coupled signalling pathways are the predominant mode of opioid adaptation [62]. In addition to adaptations at the receptor level, recent studies have indicated that endogenous neuropeptides may also modulate the effects of morphine and endogenous opioid peptides [64,65]. Administration of morphine results in the release of anti-opioid peptides that attenuate the effects of morphine. Hence, anti-opioid peptides may participate in morphine tolerance[63]. It has been demonstrated that chronic treatment with an opioid antagonist resulted in a reduction of morphine tolerance [66]. Interestingly, preproenkephalin KO mice also show reduced morphine tolerance [32]. Therefore, the deficiency of endogenous enkephalin in KO mice may alter the endogenous anti-opioid system, in turn enhancing the effect of exogenous morphine. The enhancement of morphine-induced analgesia in KO mice, as observed in the present study, indicates that there is a potential antagonistic interaction between the endogenous enkephalinergic system and exogenous opioid analgesic action occurring in supraspinal brain structures.

## Conclusion

The present findings are consistent with the view that the S1 and ACC play different roles in pain signal processing and that the endogenous opioid system is regulated differently in these two brain regions. In the sensory-discriminative aspects of pain, distinctive intra-cortical synaptic currents in the S1 may mediate specific nociceptive information regarding the intensity and location of pain. Both the spinal and cortical pathways make it possible for S1 to integrate information from nociceptive A-delta and C fiber inputs. In the affective aspect of pain signal processing, our CSD analysis results indicate that intra-cortical synaptic currents in the ACC are different from activated currents in the S1. The cingulate cortical layer II/III sink currents initiate an intracortical, polysynaptic excitation which may relay nociceptive information to other cortical and subcortical structures. The endogenous enkephalin system was not involved in acute nociceptive transmission in the spinal cord, and in the S1 and ACC. However, morphine preferentially suppressed the supraspinal related nociceptive behavior in KO mice. This effect was evident in the potentiated differential effects of morphine on the S1 and ACC in KO mice. This potentiation may be due to the up-regulation of opioid receptors. This study supports a potential antagonistic interaction between the endogenous enkephalinergic system and exogenous opioid analgesic action in supraspinal brain structures.

## Methods

### Animals

Preproenkephalin KO mice (B6.129-Penk-rstm1Pig; background strain C57BL/6J) were purchased from Jackson Laboratories (Bar Harbor, ME, USA). Homozygous mutant offsprings were bred and polymerase chain reaction (PCR) was used to confirm the genotype of the homozygous Penk-/- mice. Using the primers, Penk common-E31, Penk WT-E1R, and Penk KO-neoRL, a 700-bp and 500-bp fragments were amplified from WT and KO mice respectively. The 700-bp band was specific to WT mice while the 500-bp band was specific in KO mice. B6.129-Penk-rstm1Pig (KO) and control C57BL/6J (WT) mice (25–35 g body weight) were housed in groups of five in a room with a 12 h light/dark cycle at 22°C with free access to food and water. All experiments were carried out in accordance with the guidelines established by the Academia Sinica Institutional Animal Care and Utilization Committee. Efforts were made to minimize animal suffering and reduce the number of animals used.

### Tail-withdrawal test

Mice were restrained gently in a cotton cloth bag and half of the tail was immersed from the tip in water (49°C). The latency of tail-withdrawal was determined from three trials with 30 s intervals between each measurement.

### Hot plate test

A metal hot-plate was maintained at 53 ± 0.5°C. The time to when the mouse first exhibited nocifensive behaviour (flicked or licked its hind paw) was determined. The cut-off time was 60 s for the first sign of nocifensive behavior.

### Von Frey test

A von Frey filament was attached to a force transducer (Model 1601C, IITC, Woodland Hills, CA). The mice were placed in a hanging cage with a mesh wire floor and the von Frey filament was applied against the plantar surface of the hindpaw with increasing force until the filament started to bend and the paw was withdrawn. A digital readout showed the final force before the paw withdrawal. This value was taken as the threshold of mechanical nociception (von Frey response). The threshold of the von Frey response was determined from the mean of ten trials.

### Effect of morphine on tail-withdrawal and hot-plate response

Morphine was administrated intraperitoneally (5 or 10 mg/kg) after testing the baseline response for the hot-plate test. To study the time course of the effect of morphine, the hot-plate latency was recorded 10, 25, 40 and 55 min after morphine injection and then converted to % MPE. % MPE = (post-stress latency - baseline latency)/(cut-off time - baseline latency)*100.

### Surgical operation for electrophysiological measurements

Mice were initially anesthetized with 4% halothane (in 100% O_2_) in an acrylic box. Mice were then anesthetized with 2% halothane (in 100% O_2_) for the duration of the surgery. Body temperature was maintained at a minimum of 36.5°C via a homeothermic blanket system (Model 50-7079, Harvard Apparatus, USA). A craniotomy was performed over the skull regions covering the S1 and ACC regions. Small parts of the dura over S1 and ACC were carefully removed using a 23-gauge needle. Warm paraffin was applied to keep the cortical surface moist. Electrocardiographs were performed to monitor the heart rate. After surgical preparation, animals were anesthetized with 0.75 – 1.0% halothane and a mixture of nitrous oxide/oxygen during the recording session. The depth of anaesthesia was checked and maintained periodically by pinching the tail so that no overt body movement or acceleration of the heart rate was observed.

### Electrical stimulation

Two 23-gauge needles were inserted into the hind paw and used to deliver bipolar electrical stimulation (0.3 – 10 mA, 0.5 ms duration, 0.1 Hz) by an isolated pulse stimulator (Model 2100, A-M System Inc., USA). The anode was placed about 4 mm distal to the cathode.

### Laser stimulation

The laser pulse was generated from a surgical CO_2 _laser (Model 20 CH, Direct Energy Inc., CA, USA) and produced a radiation beam in the infrared (10.6 mm wavelength). The maximum power output was 20 W and the pulse duration was adjustable. A built-in calibration system measured the peak power of laser pulses. A hand-held laser probe was used for directing the beam. During the laser stimulation, the experimenter held the laser probe and projected the laser beam to targets on the hind paw. Skin of the hind digits, paw and heel was stimulated with four pulses at a frequency of 0.9 Hz (10 W, 5 ~20 ms duration). An averaged electrophysiological recording based on 20 stimulations was obtained and no visible damage to the skin was observed. A delay of at least 15 min was taken before the same skin site was stimulated again.

### Recording evoked field potentials in the S1 and ACC

Extra-cellular field potentials evoked by the electrical pulses described above were mapped first in the S1 region (~1 mm posterior and 3 mm lateral to bregma). The position resulting in maximal positive field potential responses to hind paw electrical stimulation was located and designated as the insertion point for the Michigan probe (16 contact points, 150 *μ*m interval spacing). The probe was inserted perpendicular to the cortical surface. Another Michigan probe was used to record the extracellular field potentials in the ACC (~2.5 mm anterior and 1 mm lateral to bregma; probe inserted 40° from vertical). An Ag-AgCl reference electrode was placed in the nasal cavity. The sampling rate of recorded analog signals was 6 kHz and data were processed using a multichannel data acquisition system (TDT Inc., USA) and a personal computer.

### Current source densities method

A five-point formula [27,67] was adopted for the time span and sampling variations in each recording session in order to smooth the spatial sampling variability. The extracellular current, *I*_*m *_was derived from the second spatial derivations of the extracellular field potentials, *φ*, and was calculated with the finite difference formula:

$$I_{m}=-(1/kh^{2})\sum_{m=-n}^{n}a_{m}\phi (x+mh),$$

where h is the distance between successive measuring points (150 *μ*m in the present investigation), and x is the coordinate perpendicular to the cortical layer. The remaining constants are as follows: n = 2, k = 4, a_0 _= -2, a_± 1 _= 0 and a_± 2 _= 1.

### Recording of electromyograms on the hind leg

Bipolar stainless steel hook electrodes were inserted into the gluteus maximus and biceps femoris muscles respectively. Signals with 5 kHz sampling rate and 10 Hz high pass were obtained using a Cyberamp 380 system (Axon Inc., USA), 1202 AD conversion card (ICP DAS Inc., Taiwan) and a custom-designed acquisition program using Borland C++ Builder (National Tsing-Hua University, Taipei, Taiwan). Summation of rectified EMGs was performed off-line using a data processing program in Matlab (The MathWorks, Inc., USA).

### Drugs administration

Morphine (5 mg/kg, 10 mg/kg and 20 mg/kg) and naloxone (0.7 mg/kg) were dissolved in physiological saline and administrated by intraperitoneal injection. SKF 97541 (0.3 mg/kg, TOCRIS, UK) and CGP 55845 (10 mg/kg, TOCRIS, UK) were dissolved in saline solution and administrated by intraperitoneal injection.

### Verification of electrode placement

At the end of the experiment, a small lesion was made by passing an anodal current (30 *μ*A, 5 s) to the deepest electrode of the Michigan probe. Another lesion was made at the same lead after the Michigan probe was withdrawn by 1000 *μ*m. The brains were fixed by perfusion with normal saline followed by 10 % formalin. The brains were sliced in 50-*μ*m-thick coronal sections using a cryosection and the sections were stained with cresyl violet (Sigma, USA). Digital images of each section were obtained and showed clear electrode tracks and lesion markers in the S1 or ACC regions. The mice atlas of Paxinos & Waston [68] was used as a reference when detailed cortical layer structures were estimated. The positions of some recording points were estimated by determining their distance from two lesion locations.

### Data Analysis

Based on the CSD data analysis in our previous study [21], sink or source currents were identified by their relatively prominent presence in specific locations at specific cortical depths and based on their consistency across different animals. Two distinct components of the sink currents were evoked by laser pulses, and were identified in the S1 with early and late latencies respectively. Three separate components of the sink currents were evoked by electrical stimuli, and were identified at different cortical depths and different latencies in the ACC. The peak latencies and amplitudes of sink and source currents evoked by the electrical stimuli and laser pulses were determined from CSD data in individual animals. The data recorded in behavioral and electrophysiological experiments were obtained from WT mice and KO mice and from different treatment groups. The statistical significance of changes after drug application was determined using the Student's t-test and ANOVA analysis. Turkey's post hoc tests were used to detect the sources of group differences revealed by the ANOVAs. P < 0.05 was considered statistically significant.

### Immunohistochemistry

Anesthetized animals (WT and KO, n = 6 per group) were perfused with 0.9% NaCl and subsequently with 4% paraformaldehyde in 0.1 M phosphate buffered saine (PBS, pH 7.4). The brains were removed, post fixed for 72 h, sectioned at 50 *μ*m on a cryosection and processed for immunohistochemistry. The sections were incubated with normal goat serum for 1 h and then reacted with rabbit polyclonal anti-met-enkephalin antibody (AB5026, Chemicon, USA) and anti-*μ*-opioid receptor antibody (AB5511, Chemicon, USA), at dilutions of 1:200 and 1:2000 respectively for 24 h at 4°C. Immunohistochemistry was performed using the avidin-biotin elite solution for 1 h (ABC kit, Vector Laboratories, CA). Staining was visualized using 0.03% 3,3-diaminobenzidine (DAB) and 0.07% H_2_O_2 _in Tris buffer (pH 7.4).

### Western blotting

All brain tissue lysis steps were completed at 4°C. Tissue samples were homogenized in 100 *μ*L ice cold lysis buffer with 1× protease inhibitor. The samples were lysed for 1 h on ice with occasional tapping. Samples were then centrifuged at 20,000 g for 30 min at 4°C. The supernatant was removed and the pellet left behind. The concentration of protein in the pellet was measured using a spectrophotometer. Protein samples were diluted to the same concentration and run on SDS-PAGE. After transferring, the membrane was blocked with 4% skim milk in TBS, and incubated at room temperature for at least 1 h or at 4°C overnight. The membrane was washed with TBST (TBS with 0.1% Tween-20) three times. The primary antibody solution was added in TBS with 0.5% skim milk, incubated at room temperature for 1 h (antibody titer = 1:1000 ~1: 2500). The membrane was washed with TBST three times. The secondary antibody solution (anti-rabbit IgG) was added in TBS with 0.5% skim milk, incubated at room temperature for 1 h (antibody titer = 1:5000). The membrane was washed three times with TBST and soaked in BCIP/NBT substrate solution. After the color developed, the membrane was washed with H_2_O_2 _to stop the reaction and then let dry.

## Abbreviations

KO: Pre-proenkephalin knock out mice; WT: Wild type mice; *ppENK*: Pre-proenkephalin; S1: Primary somatosensory cortex; ACC: Anterior cingulate cortex; CSD: Current source density; PCR: Polymer chain reaction.

## Competing interests

The authors declare that they have no competing interests.

## Authors' contributions

TCC participated in the design of the study, conducted the experiments, analyzed the data and drafted the manuscript. YYC participated in the initial part of experiments. WZS participated in discussion of the experimental results and suggestions of experiments. BCS conceived the study, participated in its design and coordination and in the writing of the manuscript. All authors read and approved the final manuscript.
